# {2,6-Bis[(di-*tert*-butyl­phosphino)­methyl]­phenyl}chloridonickel(II)

**DOI:** 10.1107/S1600536808029814

**Published:** 2008-09-20

**Authors:** Brian J. Boro, Diane A. Dickie, Karen I. Goldberg, Richard A. Kemp

**Affiliations:** a Department of Chemistry and Chemical Biology, MSC03 2060, 1 University of New Mexico, Albuquerque, NM 87131, USA; b Department of Chemistry and Biochemistry, University of Washington, Seattle, WA 98195, USA

## Abstract

In the title compound, [Ni(C_24_H_43_P_2_)Cl], the Ni atom adopts a distorted square-planar geometry, with the P atoms of the 2,6-bis­[(di-*tert*-butyl­phosphino)meth­yl]phenyl ligand *trans* to one another. The P—Ni—P plane is twisted out of the plane of the aromatic ring by 21.97 (6)°.

## Related literature

For the original synthesis and spectroscopic characterization of the title compound, see: Moulton & Shaw (1976 ▶). For the crystallographic characterization of the Pd analogue, see: Kimmich *et al.* (2002 ▶). For crystallographic characterization of the 2,6-bis­[(di-*tert*-butyl­phosphino)meth­yl]benzene ligand, see: Hollink *et al.* (2003 ▶). For related literature, see: Denney *et al.* (2006 ▶); Keith *et al.* (2006 ▶).
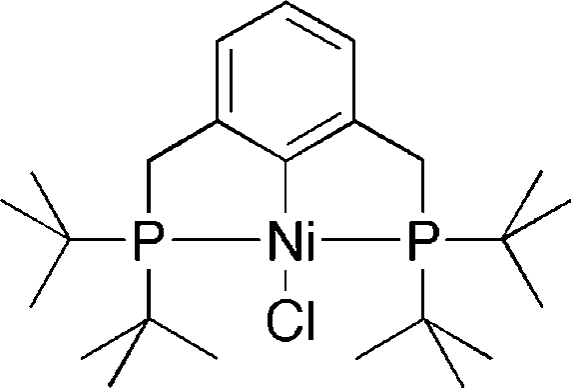


## Experimental

### 

#### Crystal data

[Ni(C_24_H_43_P_2_)Cl]
*M* *_r_* = 487.68
Orthorhombic, 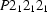

*a* = 11.3394 (4) Å
*b* = 15.0463 (5) Å
*c* = 15.4184 (5) Å
*V* = 2630.63 (15) Å^3^
*Z* = 4
Mo *K*α radiation
μ = 0.97 mm^−1^
*T* = 225 (2) K
0.50 × 0.50 × 0.40 mm

#### Data collection

Bruker SMART CCD area-detector diffractometer
Absorption correction: multi-scan (*SADABS*; Bruker, 2004 ▶) *T*_min_ = 0.622, *T*_max_ = 0.679
84881 measured reflections
10074 independent reflections
8461 reflections with *I* > 2σ(*I*)
*R*_int_ = 0.048

#### Refinement

*R*[*F* ^2^ > 2σ(*F* ^2^)] = 0.033
*wR*(*F* ^2^) = 0.084
*S* = 1.09
10074 reflections
265 parameters
H-atom parameters constrained
Δρ_max_ = 0.32 e Å^−3^
Δρ_min_ = −0.50 e Å^−3^
Absolute structure: Flack (1983 ▶), with 4507 Friedel pairs
Flack parameter: 0.006 (7)

### 

Data collection: *SMART* (Bruker, 2003 ▶); cell refinement: *SAINT* (Bruker, 2004 ▶); data reduction: *SAINT*; program(s) used to solve structure: *SHELXS97* (Sheldrick, 2008 ▶); program(s) used to refine structure: *SHELXL97* (Sheldrick, 2008 ▶); molecular graphics: *DIAMOND* (Brandenburg, 1999 ▶); software used to prepare material for publication: *publCIF* (Westrip, 2008 ▶).

## Supplementary Material

Crystal structure: contains datablocks I, global. DOI: 10.1107/S1600536808029814/pv2105sup1.cif

Structure factors: contains datablocks I. DOI: 10.1107/S1600536808029814/pv2105Isup2.hkl

Additional supplementary materials:  crystallographic information; 3D view; checkCIF report

## Figures and Tables

**Fig. 1.**
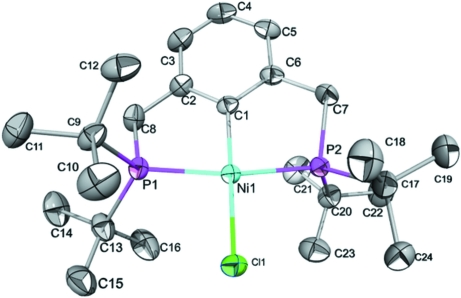
View of the title compound showing numbering scheme. Ellipsoids are shown at 50% probability and hydrogen atoms have been removed for clarity.

## supplementary crystallographic information

### Comment

The title compound, (I), was originally prepared by Moulton & Shaw (1976) but its crystal structure was not determined at that time. We have prepared (I) as part of our studies of PCP 'pincer' complexes of divalent late transition metals, which show promise as catalysts for the epoxidation of olefins (Denney *et al.*, 2006; Keith *et al.*, 2006).

In the molecular stucture of (I) (Fig. 1), the nickel adopts a square planar geometry, with the phosphorus atoms *trans* to one another. The Ni—P bond lengths 2.1921 (4) and 2.1978 (4) Å, are significantly shorter than the corresponding Pd—P bonds [2.3039 (6) and 2.3969 (6) Å] in the analogous palladium complex (Kimmich *et al.*, 2002). Steric hindrance distorts the P—Ni—P bond angle to 169.651 (18)°, while the less constrained C—Ni—Cl angle is much closer to linearity at 176.13 (5)°.

Significant geometrical changes are observed in the 2,6-bis[(di-*tert*-butylphosphino)methyl]benzene ligand upon binding to nickel. In the free ligand (Hollink *et al.*, 2003), the average P—C_methylene_ bond length is 1.870 Å, while in (I), it has decreased to 1.8308 (19) Å (P1—C8) and 1.8341 (18) Å (P2—C7). This bond shortening is accompanied by change in the P—C_methylene_—C_phenyl_ angle, from 114.5° in the free ligand to 106.23 (12)° (P1—C8—C2) and 106.84 (12)° (P2—C7—C6) in (I).

### Experimental

A solution of nickel chloride hexahydrate (0.6 g, 2.5 mmol) dissolved in 2 ml of degassed water was added to a solution of 2,6-bis-[(di-*tert*-butylphosphino)methyl]benzene (1.02 g, 2.6 mmol) in 10 ml ethanol. The solution was heated to reflux. A golden-yellow precipitate began to form only after 0.5 h. The solution was stirred under gentle reflux overnight. After cooling, the product was collected by filtration and washed with cold ethanol. It was recrystallized from a concentrated solution of pentane at 238 K.

### Refinement

Hydrogen atoms were included at geometrically idealized positions with C—H distances 0.94, 0.97 and 0.98 Å, for aryl, methyl and methylene H-atoms in a riding mode on the respective heavy atoms. The isotropic displacement parameters for the hydrogen atoms were fixed at 1.5 and 1.2 times *U*_eq_ of the parent methyl and non-methyl C-atoms. An absolute structure was determined (Flack, 1983) employing 4507 Friedel pairs of reflections which were not merged.

### Crystal data

**Table:**

[Ni(C_24_H_43_P_2_)Cl]	*F*(000) = 1048
*M**_r_* = 487.68	*D*_x_ = 1.231 Mg m^−^^3^
Orthorhombic, *P*2_1_2_1_2_1_	Mo *K*α radiation, λ = 0.71073 Å
Hall symbol: P 2ac 2ab	Cell parameters from 8656 reflections
*a* = 11.3394 (4) Å	θ = 2.2–32.5°
*b* = 15.0463 (5) Å	µ = 0.97 mm^−^^1^
*c* = 15.4184 (5) Å	*T* = 225 K
*V* = 2630.63 (15) Å^3^	Prism, gold-brown
*Z* = 4	0.50 × 0.50 × 0.40 mm

### Data collection

**Table:**

Bruker SMART CCD area-detector diffractometer	10074 independent reflections
Radiation source: fine-focus sealed tube	8461 reflections with *I* > 2σ(*I*)
graphite	*R*_int_ = 0.048
φ and ω scans	θ_max_ = 33.2°, θ_min_ = 1.9°
Absorption correction: empirical (using intensity measurements) (SADABS; Bruker, 2004)	*h* = −17→17
*T*_min_ = 0.622, *T*_max_ = 0.679	*k* = −23→23
84881 measured reflections	*l* = −23→23

### Refinement

**Table:**

Refinement on *F*^2^	Secondary atom site location: difference Fourier map
Least-squares matrix: full	Hydrogen site location: inferred from neighbouring sites
*R*[*F*^2^ > 2σ(*F*^2^)] = 0.033	H-atom parameters constrained
*wR*(*F*^2^) = 0.084	*w* = 1/[σ^2^(*F*_o_^2^) + (0.0439*P*)^2^] where *P* = (*F*_o_^2^ + 2*F*_c_^2^)/3
*S* = 1.09	(Δ/σ)_max_ = 0.002
10074 reflections	Δρ_max_ = 0.32 e Å^−^^3^
265 parameters	Δρ_min_ = −0.50 e Å^−^^3^
0 restraints	Absolute structure: Flack (1983), with 4507 Friedel pairs
Primary atom site location: structure-invariant direct methods	Flack parameter: 0.006 (7)

### Special details

**Table:**

Experimental. Yield = 60%. ^1^H NMR (250 MHz, C_6_D_6_) δ 7.00 (t, 1H, ^3^J_HH_ = 7.4 Hz, Ar-*H_para_*), 6.84 (d, 2H, ^3^J_HH_ = 7.4 Hz, Ar-*H_meta_*), 2.91 (virtual t, 4H, J_HP_ = 6.8 Hz, C*H*_2_), 1.40 (virtual t, 36H, J_HP_ = 12.7 Hz, C*H*_3_) p.p.m. ^13^C{^1^H} NMR (63 MHz, C_6_D_6_) δ 155.7 (t, ^2^J_CP_ = 16.7 Hz, Ar-*C_ipso_*), 153.0 (virtual t, J_CP_ = 25.5 Hz, Ar-*C_ortho_*), 125.2 (s, Ar-*C_para_*), 121.8 (virtual t, J_CP_ = 16.7 Hz, Ar-*C_meta_*), 34.9 (virtual t, J_CP_ = 13.4 Hz, P*C*H_2_), 34.3 (virtual t, J_CP_ = 22.7 Hz, P*C*(CH_3_)_3_), 29.8 (s, *C*H_3_) p.p.m. ^31^P{^1^H} NMR (101 MHz, C_6_D_6_) δ 66.9 p.p.m.
Geometry. All e.s.d.'s (except the e.s.d. in the dihedral angle between two l.s. planes) are estimated using the full covariance matrix. The cell e.s.d.'s are taken into account individually in the estimation of e.s.d.'s in distances, angles and torsion angles; correlations between e.s.d.'s in cell parameters are only used when they are defined by crystal symmetry. An approximate (isotropic) treatment of cell e.s.d.'s is used for estimating e.s.d.'s involving l.s. planes.
Refinement. Refinement of *F*^2^ against ALL reflections. The weighted *R*-factor *wR* and goodness of fit *S* are based on *F*^2^, conventional *R*-factors *R* are based on *F*, with *F* set to zero for negative *F*^2^. The threshold expression of *F*^2^ > σ(*F*^2^) is used only for calculating *R*-factors(gt) *etc*. and is not relevant to the choice of reflections for refinement. *R*-factors based on *F*^2^ are statistically about twice as large as those based on *F*, and *R*- factors based on ALL data will be even larger.

### Fractional atomic coordinates and isotropic or equivalent isotropic displacement parameters (Å^2^)

**Table:**

	*x*	*y*	*z*	*U*_iso_*/*U*_eq_	
Ni1	0.536566 (17)	0.968358 (13)	0.925683 (12)	0.02623 (5)	
Cl1	0.60025 (5)	1.02689 (4)	0.80121 (3)	0.04914 (12)	
P1	0.38227 (4)	0.90497 (3)	0.86644 (3)	0.02696 (8)	
P2	0.67658 (4)	1.02487 (3)	1.00738 (3)	0.02786 (8)	
C1	0.48595 (14)	0.91031 (10)	1.03045 (10)	0.0278 (3)	
C2	0.37594 (16)	0.86671 (11)	1.03569 (11)	0.0326 (3)	
C3	0.34528 (18)	0.81652 (13)	1.10805 (13)	0.0405 (4)	
H3	0.2723	0.7869	1.1094	0.049*	
C4	0.4201 (2)	0.80982 (13)	1.17718 (13)	0.0441 (5)	
H4	0.3992	0.7750	1.2254	0.053*	
C5	0.52717 (18)	0.85476 (12)	1.17589 (11)	0.0383 (4)	
H5	0.5780	0.8516	1.2240	0.046*	
C6	0.55930 (16)	0.90460 (11)	1.10322 (11)	0.0327 (3)	
C7	0.67683 (17)	0.95101 (13)	1.10201 (12)	0.0391 (4)	
H7A	0.7409	0.9076	1.0974	0.047*	
H7B	0.6879	0.9854	1.1554	0.047*	
C8	0.29202 (16)	0.87651 (14)	0.96090 (12)	0.0382 (4)	
H8A	0.2345	0.9236	0.9727	0.046*	
H8B	0.2494	0.8208	0.9508	0.046*	
C9	0.41665 (16)	0.79598 (10)	0.81364 (12)	0.0339 (3)	
C10	0.5097 (2)	0.80785 (14)	0.74313 (14)	0.0488 (5)	
H10A	0.5784	0.8371	0.7674	0.073*	
H10B	0.4776	0.8439	0.6966	0.073*	
H10C	0.5322	0.7501	0.7205	0.073*	
C11	0.3068 (2)	0.75117 (14)	0.77561 (15)	0.0497 (5)	
H11A	0.2776	0.7861	0.7273	0.075*	
H11B	0.2463	0.7471	0.8199	0.075*	
H11C	0.3271	0.6920	0.7556	0.075*	
C12	0.4694 (2)	0.73571 (12)	0.88360 (14)	0.0471 (5)	
H12A	0.4934	0.6798	0.8577	0.071*	
H12B	0.4108	0.7245	0.9281	0.071*	
H12C	0.5374	0.7646	0.9093	0.071*	
C13	0.28236 (17)	0.97553 (14)	0.79810 (13)	0.0422 (4)	
C14	0.15386 (19)	0.94355 (18)	0.79892 (18)	0.0618 (6)	
H14A	0.1057	0.9840	0.7651	0.093*	
H14B	0.1252	0.9420	0.8582	0.093*	
H14C	0.1494	0.8844	0.7740	0.093*	
C15	0.3241 (2)	0.98364 (15)	0.70379 (13)	0.0539 (5)	
H15A	0.3162	0.9266	0.6751	0.081*	
H15B	0.4061	1.0020	0.7028	0.081*	
H15C	0.2764	1.0275	0.6738	0.081*	
C16	0.2868 (3)	1.06881 (15)	0.83988 (18)	0.0642 (7)	
H16A	0.2328	1.1081	0.8098	0.096*	
H16B	0.3663	1.0923	0.8357	0.096*	
H16C	0.2643	1.0646	0.9004	0.096*	
C17	0.83287 (16)	1.01907 (13)	0.96814 (12)	0.0369 (4)	
C18	0.84152 (19)	0.92899 (15)	0.92080 (17)	0.0524 (5)	
H18A	0.9217	0.9200	0.9007	0.079*	
H18B	0.7882	0.9288	0.8716	0.079*	
H18C	0.8201	0.8815	0.9604	0.079*	
C19	0.92396 (18)	1.02033 (17)	1.04173 (15)	0.0514 (5)	
H19A	1.0020	1.0103	1.0180	0.077*	
H19B	0.9055	0.9739	1.0832	0.077*	
H19C	0.9219	1.0776	1.0705	0.077*	
C20	0.63680 (18)	1.13804 (12)	1.04915 (13)	0.0403 (4)	
C21	0.5238 (2)	1.12685 (15)	1.10274 (17)	0.0593 (6)	
H21A	0.5401	1.0901	1.1530	0.089*	
H21B	0.4636	1.0987	1.0674	0.089*	
H21C	0.4962	1.1847	1.1217	0.089*	
C22	0.7314 (2)	1.17943 (16)	1.10738 (16)	0.0585 (6)	
H22A	0.8020	1.1907	1.0735	0.088*	
H22B	0.7500	1.1389	1.1543	0.088*	
H22C	0.7023	1.2349	1.1312	0.088*	
C23	0.6101 (3)	1.19968 (14)	0.97333 (16)	0.0571 (6)	
H23A	0.5786	1.2553	0.9951	0.086*	
H23B	0.5527	1.1719	0.9354	0.086*	
H23C	0.6821	1.2111	0.9412	0.086*	
C24	0.86278 (18)	1.09373 (16)	0.90427 (14)	0.0491 (5)	
H24A	0.8597	1.1505	0.9340	0.074*	
H24B	0.8062	1.0936	0.8571	0.074*	
H24C	0.9414	1.0844	0.8812	0.074*	

### Atomic displacement parameters (Å^2^)

**Table:**

	*U*^11^	*U*^22^	*U*^33^	*U*^12^	*U*^13^	*U*^23^
Ni1	0.02964 (9)	0.02516 (9)	0.02389 (9)	−0.00470 (8)	−0.00320 (8)	0.00305 (7)
Cl1	0.0605 (3)	0.0569 (3)	0.02997 (19)	−0.0283 (3)	−0.00855 (19)	0.0151 (2)
P1	0.02591 (18)	0.02588 (17)	0.02911 (19)	−0.00090 (15)	−0.00127 (15)	−0.00323 (15)
P2	0.03048 (18)	0.02878 (18)	0.02431 (17)	−0.00396 (16)	−0.00252 (14)	0.00080 (16)
C1	0.0318 (8)	0.0261 (6)	0.0256 (7)	−0.0007 (6)	0.0043 (6)	0.0008 (6)
C2	0.0343 (8)	0.0312 (8)	0.0321 (8)	−0.0005 (7)	0.0090 (7)	−0.0036 (6)
C3	0.0416 (10)	0.0390 (9)	0.0411 (9)	−0.0050 (8)	0.0187 (8)	−0.0025 (8)
C4	0.0579 (12)	0.0399 (9)	0.0345 (9)	0.0028 (9)	0.0192 (9)	0.0076 (7)
C5	0.0465 (10)	0.0410 (9)	0.0273 (8)	0.0087 (8)	0.0051 (8)	0.0062 (7)
C6	0.0394 (9)	0.0319 (8)	0.0268 (7)	0.0021 (7)	0.0030 (6)	0.0027 (6)
C7	0.0393 (9)	0.0472 (10)	0.0309 (8)	−0.0033 (8)	−0.0079 (7)	0.0094 (7)
C8	0.0301 (8)	0.0469 (10)	0.0377 (9)	−0.0050 (7)	0.0061 (7)	−0.0095 (8)
C9	0.0396 (9)	0.0247 (7)	0.0375 (9)	−0.0030 (6)	0.0075 (7)	−0.0057 (6)
C10	0.0569 (13)	0.0432 (10)	0.0465 (11)	0.0010 (9)	0.0210 (9)	−0.0067 (8)
C11	0.0562 (12)	0.0374 (10)	0.0556 (12)	−0.0123 (9)	0.0036 (10)	−0.0146 (9)
C12	0.0604 (12)	0.0281 (8)	0.0529 (11)	0.0085 (9)	0.0101 (11)	0.0009 (7)
C13	0.0421 (9)	0.0404 (9)	0.0442 (10)	0.0116 (8)	−0.0140 (8)	−0.0054 (9)
C14	0.0352 (10)	0.0807 (17)	0.0696 (15)	0.0119 (10)	−0.0166 (10)	−0.0113 (13)
C15	0.0648 (14)	0.0528 (12)	0.0441 (11)	0.0079 (11)	−0.0195 (10)	0.0053 (9)
C16	0.0812 (18)	0.0433 (11)	0.0682 (16)	0.0299 (12)	−0.0268 (14)	−0.0098 (11)
C17	0.0303 (8)	0.0426 (9)	0.0378 (9)	−0.0044 (7)	−0.0011 (7)	0.0008 (8)
C18	0.0414 (10)	0.0530 (12)	0.0627 (13)	0.0072 (9)	0.0048 (10)	−0.0128 (11)
C19	0.0348 (9)	0.0644 (13)	0.0551 (12)	−0.0068 (9)	−0.0094 (9)	0.0041 (11)
C20	0.0479 (11)	0.0351 (9)	0.0378 (9)	−0.0036 (8)	0.0014 (8)	−0.0089 (7)
C21	0.0600 (14)	0.0528 (12)	0.0650 (14)	0.0047 (11)	0.0205 (12)	−0.0166 (11)
C22	0.0662 (15)	0.0557 (13)	0.0535 (13)	−0.0148 (11)	−0.0036 (12)	−0.0227 (11)
C23	0.0776 (17)	0.0351 (10)	0.0586 (14)	0.0104 (11)	−0.0019 (13)	0.0007 (9)
C24	0.0399 (10)	0.0612 (12)	0.0462 (11)	−0.0106 (9)	0.0041 (8)	0.0102 (10)

### Geometric parameters (Å, °)

**Table:**

Ni1—C1	1.9239 (15)	C13—C15	1.534 (3)
Ni1—P1	2.1921 (4)	C13—C14	1.535 (3)
Ni1—P2	2.1978 (4)	C13—C16	1.545 (3)
Ni1—Cl1	2.2317 (5)	C14—H14A	0.9700
P1—C8	1.8308 (19)	C14—H14B	0.9700
P1—C9	1.8720 (16)	C14—H14C	0.9700
P1—C13	1.8763 (18)	C15—H15A	0.9700
P2—C7	1.8341 (18)	C15—H15B	0.9700
P2—C17	1.8746 (19)	C15—H15C	0.9700
P2—C20	1.8756 (19)	C16—H16A	0.9700
C1—C6	1.399 (2)	C16—H16B	0.9700
C1—C2	1.412 (2)	C16—H16C	0.9700
C2—C3	1.391 (2)	C17—C24	1.532 (3)
C2—C8	1.502 (3)	C17—C19	1.534 (3)
C3—C4	1.366 (3)	C17—C18	1.543 (3)
C3—H3	0.9400	C18—H18A	0.9700
C4—C5	1.390 (3)	C18—H18B	0.9700
C4—H4	0.9400	C18—H18C	0.9700
C5—C6	1.397 (2)	C19—H19A	0.9700
C5—H5	0.9400	C19—H19B	0.9700
C6—C7	1.505 (3)	C19—H19C	0.9700
C7—H7A	0.9800	C20—C23	1.523 (3)
C7—H7B	0.9800	C20—C22	1.532 (3)
C8—H8A	0.9800	C20—C21	1.534 (3)
C8—H8B	0.9800	C21—H21A	0.9700
C9—C10	1.525 (3)	C21—H21B	0.9700
C9—C12	1.531 (3)	C21—H21C	0.9700
C9—C11	1.533 (3)	C22—H22A	0.9700
C10—H10A	0.9700	C22—H22B	0.9700
C10—H10B	0.9700	C22—H22C	0.9700
C10—H10C	0.9700	C23—H23A	0.9700
C11—H11A	0.9700	C23—H23B	0.9700
C11—H11B	0.9700	C23—H23C	0.9700
C11—H11C	0.9700	C24—H24A	0.9700
C12—H12A	0.9700	C24—H24B	0.9700
C12—H12B	0.9700	C24—H24C	0.9700
C12—H12C	0.9700		
			
C1—Ni1—P1	85.08 (5)	C15—C13—C14	109.01 (18)
C1—Ni1—P2	84.83 (5)	C15—C13—C16	108.2 (2)
P1—Ni1—P2	169.651 (18)	C14—C13—C16	108.22 (19)
C1—Ni1—Cl1	176.13 (5)	C15—C13—P1	113.01 (14)
P1—Ni1—Cl1	94.109 (18)	C14—C13—P1	113.03 (17)
P2—Ni1—Cl1	96.109 (17)	C16—C13—P1	105.07 (13)
C8—P1—C9	104.92 (9)	C13—C14—H14A	109.5
C8—P1—C13	103.98 (9)	C13—C14—H14B	109.5
C9—P1—C13	112.16 (9)	H14A—C14—H14B	109.5
C8—P1—Ni1	102.50 (6)	C13—C14—H14C	109.5
C9—P1—Ni1	113.34 (6)	H14A—C14—H14C	109.5
C13—P1—Ni1	118.02 (7)	H14B—C14—H14C	109.5
C7—P2—C17	103.12 (9)	C13—C15—H15A	109.5
C7—P2—C20	106.10 (9)	C13—C15—H15B	109.5
C17—P2—C20	112.36 (9)	H15A—C15—H15B	109.5
C7—P2—Ni1	102.87 (6)	C13—C15—H15C	109.5
C17—P2—Ni1	118.68 (6)	H15A—C15—H15C	109.5
C20—P2—Ni1	111.98 (7)	H15B—C15—H15C	109.5
C6—C1—C2	116.79 (15)	C13—C16—H16A	109.5
C6—C1—Ni1	121.59 (12)	C13—C16—H16B	109.5
C2—C1—Ni1	121.51 (12)	H16A—C16—H16B	109.5
C3—C2—C1	121.26 (17)	C13—C16—H16C	109.5
C3—C2—C8	120.69 (17)	H16A—C16—H16C	109.5
C1—C2—C8	118.05 (15)	H16B—C16—H16C	109.5
C4—C3—C2	120.70 (18)	C24—C17—C19	108.50 (16)
C4—C3—H3	119.6	C24—C17—C18	109.02 (17)
C2—C3—H3	119.6	C19—C17—C18	108.54 (18)
C3—C4—C5	119.69 (17)	C24—C17—P2	112.50 (14)
C3—C4—H4	120.2	C19—C17—P2	113.40 (13)
C5—C4—H4	120.2	C18—C17—P2	104.70 (13)
C4—C5—C6	120.04 (18)	C17—C18—H18A	109.5
C4—C5—H5	120.0	C17—C18—H18B	109.5
C6—C5—H5	120.0	H18A—C18—H18B	109.5
C5—C6—C1	121.42 (16)	C17—C18—H18C	109.5
C5—C6—C7	119.36 (17)	H18A—C18—H18C	109.5
C1—C6—C7	119.21 (14)	H18B—C18—H18C	109.5
C6—C7—P2	106.84 (12)	C17—C19—H19A	109.5
C6—C7—H7A	110.4	C17—C19—H19B	109.5
P2—C7—H7A	110.4	H19A—C19—H19B	109.5
C6—C7—H7B	110.4	C17—C19—H19C	109.5
P2—C7—H7B	110.4	H19A—C19—H19C	109.5
H7A—C7—H7B	108.6	H19B—C19—H19C	109.5
C2—C8—P1	106.23 (12)	C23—C20—C22	109.96 (19)
C2—C8—H8A	110.5	C23—C20—C21	108.3 (2)
P1—C8—H8A	110.5	C22—C20—C21	108.32 (18)
C2—C8—H8B	110.5	C23—C20—P2	109.70 (13)
P1—C8—H8B	110.5	C22—C20—P2	113.70 (16)
H8A—C8—H8B	108.7	C21—C20—P2	106.63 (13)
C10—C9—C12	107.52 (16)	C20—C21—H21A	109.5
C10—C9—C11	109.93 (16)	C20—C21—H21B	109.5
C12—C9—C11	109.06 (16)	H21A—C21—H21B	109.5
C10—C9—P1	110.57 (12)	C20—C21—H21C	109.5
C12—C9—P1	107.09 (12)	H21A—C21—H21C	109.5
C11—C9—P1	112.49 (13)	H21B—C21—H21C	109.5
C9—C10—H10A	109.5	C20—C22—H22A	109.5
C9—C10—H10B	109.5	C20—C22—H22B	109.5
H10A—C10—H10B	109.5	H22A—C22—H22B	109.5
C9—C10—H10C	109.5	C20—C22—H22C	109.5
H10A—C10—H10C	109.5	H22A—C22—H22C	109.5
H10B—C10—H10C	109.5	H22B—C22—H22C	109.5
C9—C11—H11A	109.5	C20—C23—H23A	109.5
C9—C11—H11B	109.5	C20—C23—H23B	109.5
H11A—C11—H11B	109.5	H23A—C23—H23B	109.5
C9—C11—H11C	109.5	C20—C23—H23C	109.5
H11A—C11—H11C	109.5	H23A—C23—H23C	109.5
H11B—C11—H11C	109.5	H23B—C23—H23C	109.5
C9—C12—H12A	109.5	C17—C24—H24A	109.5
C9—C12—H12B	109.5	C17—C24—H24B	109.5
H12A—C12—H12B	109.5	H24A—C24—H24B	109.5
C9—C12—H12C	109.5	C17—C24—H24C	109.5
H12A—C12—H12C	109.5	H24A—C24—H24C	109.5
H12B—C12—H12C	109.5	H24B—C24—H24C	109.5
